# The clinical importance of measuring glycaemic variability: Utilising new metrics to optimise glycaemic control

**DOI:** 10.1111/dom.16098

**Published:** 2024-12-05

**Authors:** R. A. Ajjan

**Affiliations:** ^1^ LIGHT Laboratories, Leeds Institute for Cardiovascular and Metabolic Medicine University of Leeds Leeds UK

**Keywords:** continuous glucose monitoring, glycaemic variability, hypoglycaemia, macrovascular complications, microvascular complications, patient‐related outcome measures, type 1 diabetes, type 2 diabetes

## Abstract

With the widespread use of continuous glucose monitoring (CGM), glycaemic variability (GV) is a glucose metric that has been gaining increasing attention. However, unlike other glucose metrics that are easily defined and have clear targets, GV has a large number of different measures given the complexity involved in assessment. While variabilities in HbA1c, fasting and postprandial glucose have been incorporated under the GV banner, short‐term variability in glucose, within day and between days, is more in keeping with the correct definition of GV. This review is focused on short‐term GV, as assessed by CGM data, although studies calculating GV from capillary glucose testing are also mentioned as appropriate. The different measures of GV are addressed, and their potential role in microvascular and macrovascular complications, as well as patient‐related outcomes, discussed. It should be noted that the independent role of GV in vascular pathology is not always clear, given the inconsistent findings in different populations and the close association between GV and hypoglycaemia, itself an established risk factor for adverse outcomes. Therefore, this review attempts, where possible, to disentangle the contribution of GV to diabetes complications from other glycaemic parameters, particularly hypoglycaemia. Evidence to date strongly suggests an independent role for GV in vascular pathology but future large‐scale outcome studies are required to fully understand the exact contribution of this metric to vascular complications. This can be followed by setting appropriate GV measures and targets in different diabetes subgroups, in order to optimise glycaemic management and limit the risk of complications.

## Abstract 2

Please visit https://health.learning.wiley.com/courses/dom-16098/ to complete an accredited learning activity for this article and receive 1.0 *AMA PRA Category 1 Credit*™.

## 1 INTRODUCTION

HbA1c has been the main metric in use to assess glycaemic control given the association with diabetes complications in both type 1 and type 2 diabetes.^1^, ^2^, ^3^, ^4^ However, it is now accepted that HbA1c only offers partial assessment of glucose control and additional glycaemic markers are required for the comprehensive evaluation of glycaemia.^5^ Previous work has shown that lower HbA1c does not necessarily translate into better outcomes, indicating the presence of other glucose metrics that play a role.^6^, ^7^ It is worth noting that specificity of HbA1c at predicting complications is low, despite its high sensitivity, further strengthening the argument for an effect of other glucose markers.^8^ Indeed, both hypoglycaemia and glycaemic variability (GV), which HbA1c fails to capture, have shown associations with adverse clinical outcome.^9^, ^10^ Consequently, research efforts have focused on evaluating the risk of other glycaemic factors, an area that has accelerated recently by the advent of more widely accessible continuous glucose monitoring (CGM). A comprehensive set of glycaemic data is provided by CGM, which are now routinely used in clinical management with specific suggested targets.^11^ One of these measures is GV that was rarely assessed in routine care previously but is now gaining more attention due to the association with adverse clinical outcomes (discussed below). Of note, GV shows a relationship with hypoglycaemia,^12^, ^13^, ^14^ which makes disentangling the exact role of each in predisposition to diabetes complications problematic. Moreover, unlike hypoglycaemia, which has a clear definition, GV has used different measurements to define, adding another layer of complexity to understanding its role in diabetes complications.

While a number of good quality reviews attempted to address the role of GV in diabetes complications,^15^, ^16^, ^17^ this is a quickly developing area with frequent updates required. Moreover, there is a general lack of review articles analysing the association between GV and patient‐related outcome measures (PROMs), an area that is gaining more interest in both research and clinical practice.^18^, ^19^ The current narrative review offers the reader the unique opportunity to understand the relationship between GV and diabetes complications as well as PROMs. The review also suggests future steps to better imbed GV into clinical practice, while also highlighting gaps in knowledge and areas for future research.

## 2 SEARCH STRATEGY AND STUDY SELECTION

A search was conducted across the abstracting and indexing databases: Embase and MEDLINE encompassing publication years between January 2012 and September 2024. The search terms centred on GV combined with diabetes and quality of life (QoL) outcomes as summarised in Table S1.

## 3 GV: FACTS AND DIFFICULTIES

As alluded to earlier, a key difficulty in assessing the independent effects of GV on outcomes is the close association with hypoglycaemia. High glucose levels can lead to hypoglycaemia due to correction of elevated glucose in insulin‐treated individuals with diabetes. Conversely, hypoglycaemia frequently results in subsequent hyperglycaemia due to overcorrection of low glucose levels, which increases GV. Hypoglycaemia is associated with a number of abnormalities that contribute to vascular risk including arrythmias, endothelial cell dysfunction, increased inflammation and an enhanced thrombotic environment,^20^, ^21^, ^22^ explaining the association between abnormally low glucose levels and short‐term, as well as longer‐term, cardiovascular complications and mortality.^9^, ^23^, ^24^, ^25^, ^26^ Thus, it is important to differentiate between the adverse clinical effects of GV through increased hypoglycaemia or as a truly independent risk factor. Studies including individuals without diabetes can be particularly helpful in differentiating the role of hypoglycaemia from GV, given that hypoglycaemia is less of an issue with this group. However, CGM is rarely employed in individals without diabetes and GV is usually measured using infrequent capillary glucose testing,^27^ which may question study conclusions.

**FIGURE 1:**
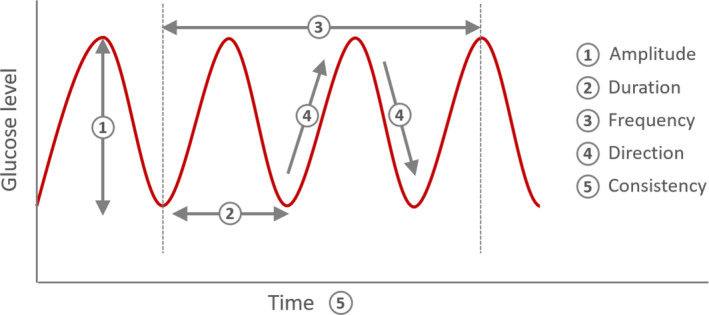
The five main dimensions of glycaemic variability (GV). These different dimensions explain that existence of a large number of GV metrics.

The other difficulty is related to the large number of GV markers studied to date. Unlike hyperglycaemic and hypoglycaemic markers, which are limited in number and are clearly defined, there are over 20 different GV markers, some of which require complicated calculations, given the different dimensions of this glycaemic metric, which can be summarised as follows (Figure 1):

1. Direction: an increase or a decrease in glucose levels.
2. Amplitude: distance between peaks and troughs of glycaemic excursions.
3. Duration: length of time over changes in glucose levels (before levels stabilise).
4. Frequency: number of fluctuations in glucose levels over a specified period of time.
5. Consistency: between days‐consistency of the changes in glucose levels.

Confusingly, the term GV has also been used to describe fluctuation in HbA1c or even fasting glucose levels over a longer period,^28^ which are not real measures of GV. Longer term variability in HbA1c is more in keeping with alterations in average glycaemic control than true GV and, therefore, this review is focused on understanding short‐term within‐day and between‐day variability in glucose levels with adjustments for the occurrence of hypoglycaemia when possible. Biomarkers have also been proposed as a measure of GV such as plasma levels of 1,5‐anhydroglucitol arguing that lower levels are an indicator of postprandial hyperglycaemic excursions.^29^, ^30^ However, 1,5‐anhydroglucitol levels correlate negatively with hyperglycaemia, and therefore, this is mainly a measure of glucose fluctuation in one direction and does not give a full picture of GV.

In general, attention is given to studies using CGM to assess fluctuation in glucose levels, given that even frequent 7–10‐point SMBG testing is not enough to reliably estimate GV.^31^, ^32^ However, some key studies using capillary glucose testing to assess GV are mentioned.

## 4 MEASURES OF GV IN CURRENT CLINICAL USE

The commonly used metrics, standard deviation (SD) and coefficient of variation (CoV) are reflections of dispersion of glucose data. A criticism of SD is its modulation by high glucose levels, although it can be argued this is a strength as it gives information on the interplay between GV and hyperglycaemia. In contrast, CoV corrects for high glucose levels, thus giving a more accurate reflection of GV per se. However, CoV can ‘improve’ simply by rising glucose levels, and therefore, it can be flawed in those with consistent hyperglycaemia. Of importance, neither SD nor CoV is that effective at assessing post‐prandial excursions in glucose, which is better analysed using mean average glucose excursion (MAGE). It should be noted that MAGE measurements can differ depending on whether the ascending or descending limbs are used for calculations, and therefore, it is not as ‘objective’ as SD or CoV. Moreover, MAGE is affected by the glucose testing method as calculations from capillary glucose testing and CGM can differ.^33^ To measure both amplitude and frequency of glucose oscillations, continuous overall net glycaemic action (CONGA) is used, while glycaemic variability percentage (GVP) measures amplitude, frequency and distance.^34^

When assessing between‐day GV, mean of daily differences (MODD) clarifies changes in glucose values at the same time of the day,^35^ which is not that dissimilar to interquartile range (IQR), reflecting glucose distribution at any single time of day.^36^ GV markers in current use are summarised in Table 1.

**TABLE 1:** The main indices of glycaemic variability (GV).

Index	Units	Definition	Interpretation	Remarks
SD	mmol/L (mg/dL)	‘Standard deviation’ of mean glucose concentrations	Short‐term within‐day glucose variability	Dispersion of glucose data. Highly influenced by mean glucose (higher glucose = higher SD)
CoV	%	‘Coefficient of variation’ of mean glucose (% SD/mean glucose)	Short‐term within‐day glucose variability	Dispersion of glucose values corrected for mean glucose
IQR	mmol/L (mg/dL)	‘Interquartile range’ calculated from AGP at a given time point. It can be corrected for median glucose and presented as IQR/med (%)	Short‐term within‐day glucose variability	Measure of glucose variation at a given time point over several days
MAGE	mmol/L (mg/dL)	‘Mean amplitude of glucose excursions’ represents the difference between peaks and troughs of glucose fluctuations. Can also be corrected for mean glucose and presented as MAG/m (%)	Short‐term within‐day glucose variability	Capture mealtime glucose excursions. Calculations can be subjective and differ depending on whether ascending or descending glucose limbs are used for calculations
MAD	mmol/L (mg/dL)	‘Mean absolute difference’ of consecutive glucose values	Short‐term within‐day glucose variability	No real advantage over SD as an estimate of glycaemic variability
GVP	%	‘Glycaemic variability percentage’ is intended to capture both the amplitude and frequency of glucose oscillations	Short‐term within‐day glucose variability	A complex measure of amplitude and frequency of glucose oscillations, as well as aspects of distance travelled
MAG	mmol/L (mg/dL)	‘Mean absolute glucose’ change assesses glucose distribution at a given time point. Can be corrected for mean glucose and presented as MAG/m (%)	Short‐term within‐day glucose variability	Can differentiate between excursions of identical extent but of different duration
CONGA	mmol/L (mg/dL)	‘Continuous overall net glycaemic action’ integrates duration and degree of glucose excursions	Short‐term within‐day glucose variability	Requires complex calculations and measures amplitude and frequency of glucose oscillations
MODD	mmol/L (mg/dL)	‘Mean of daily difference’ assesses absolute difference between two values measured at the same daily time point	Short‐term between‐day glucose variability	Established measure of inter‐day glycaemic variability
*J*‐Index	Score	Calculated from mean and SD of all glucose values	Mean glucose and stability	Complex calculation, not widely used and additional value is uncertain
LBGI	Score	‘Low blood glucose index’ was originally designed to estimate hypoglycaemia risk from sparse capillary glucose readings	Risk of low glucose	While calculations can be complex, these measures aid in differentiating between variability above and below target range. Scope for above‐target readings is significantly wider than for below‐target readings, with associated implications for impact and risk
HBGI	Score	‘High blood glucose index’ was originally designed to estimate risk of hyperglycaemia from sparse capillary glucose readings	Risk of high glucose	
GFI	mmol/L (mg/dL)	‘Glucose fluctuation index’ compares differences in consecutive readings. Can be corrected for mean glucose and presented as glucose coefficient of fluctuation (GCF, %)	Short‐term within‐day glucose variability	The advantage over other GV metrics is unclear and is rarely used

*Note*: While there are other GV measures, the most common metrics used in research use are listed in the table. The main GV metrics in current clinical use are standard deviation (SD) and coefficient of variation (CoV), although these may fail to detect some glucose fluctuations of potential clinical relevance.

It should be noted that treatment targets for most glycaemic markers are close to levels observed in individuals without diabetes, which is not the case for GV. CoV is widely used in clinical practice and a target threshold of <36% was established as reflecting relatively stable glucose levels.^37^ Therefore, CoV of <36% is advocated in international guidelines,^11^ although in the healthy population without diabetes, CoV is only around 17%.^38^, ^39^ Another issue with CoV is related to the mode of calculation as within‐day CoV can differ from measuring both within and between day CoVs (i.e. total CoV),^40^ which is usual practice in clinical studies.

It should also be noted that the degree of GV can vary according to therapies used, type of diabetes and even the age of the patient. For example, individuals with type 2 diabetes (T2D) on metformin only treatment will have a very different GV compared to those on multiple daily injections (MDI) of insulin. The same applies to individuals with type 1 diabetes (T1D) who use hybrid closed loop when compared with MDI treatment. Differences are also likely comparing MDI‐treated individuals with T1D and T2D, given the former group is younger and likely to be more active. Therefore, these factors should be taken into account when assessing GV in heterogeneous populations with diabetes, which has not been routinely addressed in studies to date.

## 5 GV AND DIABETES COMPLICATIONS

Studies have investigated variability in average glucose, measured as HbA1c and/or variability in FPG as risk factors for vascular complications and/or mortality in diabetes.^41^, ^42^, ^43^, ^44^, ^45^, ^46^ However, I will focus on short‐term GV studies, in particular those using CGM, to understand the role of daily glucose changes in diabetes complications.

### 5.1 Microvascular complications

A small study of 32 patients with T1D showed higher risk of microvascular complications with increased GV, independently of HbA1c and regardless whether GD was assessed as SD, CoV or MAGE. Of interest, GV derived from capillary glucose testing failed to show an increased risk, emphasising the importance of frequent glucose measures for investigating GV.^47^

#### 5.1.1 Nephropathy

One study assessed GV as CGM‐derived SD, CoV, MAGE and CONGA and showed correlations with short‐term deterioration in renal function in 28 T2D patients undergoing percutaneous coronary intervention.^48^ In a cross‐sectional study of 173 T2D, CGM‐derived SD and MAGE, but not CoV, were associated with albuminuria with only SD remaining significant after multivariate analysis.^49^ The association between albuminuria and SD but not CoV may be due to the central role of hyperglycaemia in diabetic nephropathy, or alternatively, it may indicate an interaction between hyperglycaemia and GV (i.e. glucose fluctuations enhance the pathological effects of hyperglycaemia). A prospective study including 999 Japanese individuals with T2D performed baseline analysis of the association between GV metrics (including SD, CoV, MAGE and MODD) and microvascular complication, including retinopathy and nephropathy.^50^ Both retinopathy and nephropathy were associated with GV metrics, while hypoglycaemic exposure failed to show an association. However, GV associations with retinopathy were lost after correcting for HbA1c but remained for nephropathy, suggesting a differential effects for GV on microvascular complications in some populations, and this remains an area for future research.

#### 5.1.2 Retinopathy

An early study of 68 diabetes patients (T1D = 35 and T2D = 33), and of whom 28 had retinopathy, showed that CGM‐derived SD, CONGA and high blood glucose index (HBGI), but not MAGE, correlated with the presence of retinopathy independently of HbA1c.^51^ However, these associations disappeared after multivariate analysis and only diabetes duration remained significant; given the small number of individuals, multivariate analysis has limited power, and therefore, it is difficult to make robust conclusions. Studies on early changes in diabetic retinopathy in T1D (*n* = 37) have made a link between retinal nerve fibre layer thickness and GV as measured by both CONGA and low blood glucose index (LBGI).^52^ A separate investigation showed correlations between LBGI and retinal sensory neuropathy in 30 T1D individuals.^53^ In a large study of 3119 individuals with diabetes, retinopathy correlated with SD in T2D, but not latent autoimmune diabetes of adults (LADA).^54^ However, numbers in the LADA group were relatively small (*n* = 192) and more work is required to investigate the potential differential effects of GV in various types of diabetes.

#### 5.1.3 Neuropathy

Diabetic peripheral neuropathy (DPN) in 45 adults with T2D and well‐controlled HbA1c showed a correlation with several markers of GV, which was not seen in DPN‐free controls, with MAGE emerging as the most significant independent risk factor.^55^ A later study on 40 individuals with diabetes (13 T1D and 27 T2D) showed an association between MAGE and peripheral neuropathy measured using nerve conduction studies.^56^ In a study of 509 T2D individuals (147 with abnormal nerve conduction) undergoing 3 days CGM, SD correlated with subclinical neuropathy.^57^ HbA1c also showed a correlation with neuropathy but time in hypoglycaemia did not differ between people with or without DPN, although the DPN group had significantly more episodes of hypoglycaemia. A cross‐sectional study on 982 T2D (DPN in 197) showed higher SD, MODD and MAGE in those with DPN; importantly, MAGE showed 65% sensitivity and 76% specificity at detecting DPN at a cutoff value of 4.60 mmol/L.^58^ Using blinded CGM and regression analysis, a correlation was shown between SD, HGBI, LGBI, but not MAGE, and sural nerve conduction velocity in 304 individuals with T2D.^59^ While studies collectively show a relationship between GV and DPN, they do not always agree on the best GV measure to employ. A recent systematic review and meta‐analysis may have solved this issue by demonstrating that increased SD, MAGE and MODD are all associated with 2–3‐fold increased odds ratios for DPN.^60^

In addition to DPN, cardiac autonomic neuropathy (CAN) has also demonstrated associations with GV. LBGI was associated with cardiovascular nerve function in 44 T1D individuals,^61^ while another study of 33 T1D individuals reported correlations between R‐R variability and SD, MAGE as well as MODD.^62^ A well‐designed study of 24 individuals with T1D and 24 controls demonstrated that relatively modest increases in CoV, SD and MAGE (by 4.9%, 0.7 mmol/L and 1.4 mmol/mol, respectively) are associated with CAN.^63^ In another study of 36 T1D individuals, most measures of GV were independently associated with CAN, but multivariable and dominance analysis revealed that level 2 hypoglycaemia was a major contributor to these observations,^64^ creating doubts over the independent effects of GV. In contrast, a study of 40 individuals with diabetes showed lower CoV in those with CAN but higher CONGA, suggesting a role for hyperglycaemic fluctuations.^65^ Similar findings for CoV and CONGA were documented in 133 individuals with T1D diabetes but the relationships were lost after adjusting for known risk factors^66^; these negative findings may have been due to the younger age of the population studied.

On the other hand, GV may have an early effect on CAN in T2D. A study of 90 newly diagnosed individuals with T2D demonstrated that MAGE, recorded through 48–72 h CGM, is associated with the presence of CAN, while CoV, MODD, fasting glucose or HbA1c showed no associations.^67^ The effects of hypoglycaemia were not assessed presumably due to the population studied (newly diagnosed patients) who are yet to receive therapies that can cause hypoglycaemia. These findings are supported by a subsequent study of 94 T2D individuals showing that baroreflex sensitivity is negatively correlated with CoV and MAGE after multivariate analysis.^68^ Indeed, CGM‐derived CoV, but not SD or MAGE, was independently associated with CAN in 110 patients with inadequately controlled T2D.^69^ A recent small study of 21 individuals with T2D and established microvascular disease showed that GV, measured as SD or CoV, is associated with cardiac arrhythmias, which may be related to CAN, while no associations were found with hypoglycaemia.^70^

More recently, emerging evidence has been reporting a relationship between GV and cognitive decline, which is well summarised in a recent systematic review.^71^ The exact mechanisms are unclear but microvascular alterations through increased inflammation and oxidative stress have been implicated, while a direct effect on brain white matter has also been proposed.

Taken together, GV seems to be related to microvascular complications but there are a number of caveats to studies conducted to date. The majority were small scale studies, and therefore inadequately powered, while the cross‐sectional nature could only demonstrate a relationship but not a ‘cause–effect’. Moreover, different GV metrics were used across the studies, and therefore, future adequately powered prospective studies, both observational and interventional, are required to fully understand the role of GV in microvascular disease. Table 2 summarises the main studies linking GV to microvascular disease.

**TABLE 2:** Summary of studies investigating the relationship between microvascular, macrovascular complications and glycaemic variability (GV).

Study [ref]	Size (*n*)	Population	GV metrics	Type of study and main findings
*Microvascular complications*				
Soupal et al., 2014^47^	32	T1D	SD, CoV. MAGE	Cross‐sectional; all GV measures correlate with the presence of microvascular disease, independently of HbA1c
Nusca et al., 2015^48^	28	T2D	SD, MAGE, CONGA	Prospective; SD, MAGE, CONGA are risk factors for post‐PCI deterioration in renal function
Jin et al., 2015^49^	173	T2D	SD, CoV	Cross‐sectional; SD, but not CoV, is associated with albuminuria after multivariate analysis
Wakasugi et al., 2021^50^	999	T2D	SD, CoV, MAGE and MODD	Cross‐sectional; GV measures are associated with nephropathy after correcting for HbA1c
Sartore et al., 2013^51^	48	T1D/T2D	SD, CONGA, HBGI, MAGE	Cross‐sectional; GV markers, except MAGE, correlate with retinopathy (correlations were lost after multivariate analysis)
Picconi et al., 2017^52^	37	T1D	LBGI, CONGA	Cross‐sectional; LBGI and CONGA are independent predictors of retinal nerve fibre layer thickness
Stem MS, 2016^53^	81	T1D/control	LBGI	Cross‐sectional; LBGI is a risk factor for altered in retinal thickness
Lu et al., 2019^54^	3119	T2D/LADA	SD, CV, MAGE	Cross‐sectional; SD correlates with diabetic retinopathy in T2D (*n* = 2927) but not in LADA (*n* = 192)
Xu et al., 2014^55^	90	T2D/control	SD, MODD, MAGE	Cross‐sectional; MAGE correlates with DPN
Akaza et al., 2018^56^	40	T1D/T2D	MAGE	Cross‐sectional; MAGE is associated with the presence of DPN measured using NCS
Pan et al., 2022^57^	509	T2D	SD	Cross‐sectional; SD correlated with subclinical neuropathy, measured using NCS
Hu Y, 2018^58^	982	T2D	SD, MODD, MAGE	Cross‐sectional; GV markers correlated with DPN and MAGE showed 65% sensitivity and 76% specificity at detecting DPN
Morita et al., 2024^59^	304	T2D	SD, HGBI, LGBI, MAGE	Cross‐sectional; an association is reported between SD, HGBI, LGBI, but not MAGE, and sural nerve conduction velocity
Jaiswal et al., 2014^61^	44	T1D	LBGI	Cross‐sectional; LBGI correlates with cardiac nerve function
Iwasaki et al., 2015^62^	31	T1D	SD, MAGE, MODD	Cross‐sectional; SD, MAGE, MODD correlate with change in R‐R interval
Naaman et al., 2022^63^	48	T1D/control	SD, CoV, MAGE	Cross‐sectional; relatively small increase in SD, COV or MAGE is associated with CAN
Jun et al., 2019^64^	80	T1D	SD, CoV, MAGE, LBGI, HBGI	Cross‐sectional; while GV markers were independently associated with GV measures, this appeared to be driven by hypoglycaemic exposure
Gad et al., 2023^65^	40	T2D/T2D	CoV, CONGA	Cross‐sectional; lower CoV in those with CAN but higher CONGA (implicating hyperglycaemic fluctuations)
Christensen et al., 2020^66^	133	T1D	CoV, CONGA, MAGE	Cross‐sectional; no correlation between GV metrics and markers of neuropathy after adjusted analysis
Xu et al., 2016^67^	90	T2D	CoV, MODD, MAGE	Cross‐sectional; MAGE, but not CoV or MODD, is associated with CAN in newly diagnosed patients
Matsutani et al., 2018^68^	94	T2D	SD, CV, MAGE	Cross‐sectional; CoV, MAGE correlate with cardiac baroreflex sensitivity after multivariate analysis
Jun et al., 2015^69^	110	T2D	SD, CoV, MAGE	Cross‐sectional; CoV, but not SD or MAGE, is independently associated with CAN
Andersen et al., 2021^70^	21	T2D	SD, CoV	Prospective; SD and CoV are associated with cardiac arrythmias
*Macrovascular and cardiac complications*				
Tang et al., 2016^72^	240	T2D	SD, MAGE, MODD	Cross‐sectional; MAGE is an independent risk 10‐year Framingham risk
Su et al., 2013^73^	222	ACS	MAGE	Prospective; MAGE is an independent predictor of MACE at 12 months in ACS patients, 119 of whom had T2D
Gerbaud, 2019^10^	327	T2D	SD	Prospective; SD predicts MACE (17 months follow‐up), independently of hypoglycaemia
Takahashi et al., 2018^74^	417	ACS	MAGE	Prospective; MAGE is an independent predictor MACCE (39 months follow‐up) in ACS patients (34% with T2D)
Akirov et al., 2019^75^	8894	Surgical patients	SD, CoV	Prospective; SD and CoV were associated with longer hospitalisation and increased risk of short‐term and long‐term mortality in surgical patients (23% with diabetes). Associations were independent of recorded hypoglycaemia
Gutierrez‐Zuniga 2023^76^	213	Acute stroke	SD	Prospective; SD independently associated with mortality at 3 months following acute ischaemic stroke (30% with diabetes)
ElMalahi et al., 2022^77^	515	T1D	SD, CoV	Prospective; neither SD nor CoV showed associations with the composite outcome of microvascular and macrovascular disease and hospitalisation at 2 years (but GV groups were not matched for age or diabetes duration)
Foreman et al., 2021^78^	816	Population‐based cohort	SD, CoV	Cross‐sectional; SD and CoV showed associations with aortic stiffness in a population‐based cohort (23% with T2D)
Taya et al., 2021^79^	600	T2D	SD, CoV, MAGE, IQR, MODD	Cross‐sectional; none of the GV metrics showed associations with IMT, although associations were documented with changes in carotid tissue characteristics
Miyoshi et al., 2021^80^	25	ACS	MAJE, MODD, J‐index, HBGI and LBGI	Cross‐sectional; MODD correlates with NT‐Pro‐BNP in ACS patients (32% with diabetes)
Yokota et al., 2019^81^	100	T2D	SD	Cross‐sectional; SD correlates with HFpEF

Abbreviations: ACS, acute coronary syndrome; CAN, cardiac autonomic neuropathy; CONGA, continuous overlapping net glycaemic action; CoV, coefficient of variation; DPN, diabetic peripheral neuropathy; GV, glycaemic variability; HFpEF, heart failure with preserved ejection fraction; LADA, latent autoimmune diabetes of the adult; LBGI, low blood‐glucose index; MACCE, major adverse cardiac and cerebrovascular events; MACE, major adverse cardiac events; MAG, mean absolute glucose; MAGE, mean amplitude of glycaemic excursion; MODD, mean of daily differences; SD, standard deviation; T1D, type 1 diabetes; T2D, type 2 diabetes.

### 5.2 Macrovascular complications

In a study involving 3 days CGM, 240 T2D patients, without a history of cardiovascular disease and having well‐controlled glycaemia (HbA1c ≤7.0%; ≤53 mmol/mol), showed that SD and MAGE were associated with 10‐year risk of CVD, and regression analysis suggested MAGE was an independent risk factor.^72^ A study on 222 individuals with recent acute coronary syndrome (ACS) (*n* = 119 with diabetes), 2 days CGM demonstrated an association of MAGE with major adverse cardiac events (MACE) at 12 months,^73^ which was independent of HbA1c. A study of 327 T2D patients with ACS has shown that MACE occurred in 89 patients (27%) over 16.9 months follow‐up with SD, at a cut off of 2.70, being the strongest glycaemic metric to predict MACE.^10^ Importantly, regression analysis showed that GV was an independent risk factor for MACE after adjusting for hypoglycaemia, which, interestingly, was a separate independent risk factor for adverse outcome. One weakness of the study is calculation of SD from capillary glucose testing rather than CGM data. In a different population of 417 ACS patients (34% with diabetes), CGM‐derived MAGE was predictive of major adverse cardiac and cerebrovascular events (MACCE) over follow‐up of 39 months.^74^ In a cohort study of 8894 hospitalised surgical patients (23% with diabetes), increased GV, as measured by SD and CoV derived from capillary glucose testing, was associated with longer hospitalisation and increased risk of short‐term and long‐term mortality.^75^ Adjusting for hypoglycaemia did not affect these outcomes, suggesting that fluctuation in glucose levels, rather than hypoglycaemia itself, is responsible for these findings. In 213 individuals (30% with diabetes) and acute ischaemic stroke, capillary glucose–derived SD for 48 h was independently associated with increased mortality at 3 months.^76^ While hypoglycaemia was not investigated, this is unlikely to be a major factor given most study participants did not have diabetes.

However, not all studies show associations between GV and macrovascular complications. In one study of 515 T1D individuals followed for a period of 2 years, neither CV nor SD demonstrated a relationship with the composite end point of macrovascular, microvascular disease and hospitalisation analysed together and separately.^77^ However, GV groups were not matched for age, diabetes duration or overall glycaemic control; thus, it is difficult to make concrete conclusions.

A cross‐sectional study of 816 population‐based cohort (23% with T2D) showed a relationship between 7 days CGM‐derived GV, measured as SD and CoV, and aortic stiffness.^78^ Hypoglycaemia was not investigated, but given the majority did not have diabetes, it is unlikely that findings were driven by hypoglycaemia. In contrast, a study of 600 Japanese individuals with T2D, undergoing up to 8 days blinded CGM, failed to show an association between different GV metrics and carotid intima media thickness.^79^ However, positive associations between GV metrics and grey scale median of the carotid arteries (a proposed early marker of atherosclerosis) were observed, leading the authors to conclude that GV is associated with changes in carotid artery tissue characteristics.

While several studies attempted to link GV with cardiovascular pathology, there is a general lack of studies addressing the association between GV and cardiac dysfunction. A small study of 25 individuals with recent acute coronary syndrome (only eight with diabetes) has shown that day to day variability in glucose levels, measured as MODD, correlated with NT‐proBNP, although no correlations were found with cardiac echocardiography measurements.^80^ In 100 individuals with T2D, CGM‐derived SD was linked to heart failure with preserved ejection fraction (HFpEF),^81^ a condition that is more common in individuals with diabetes.^82^ Table 2 summarises the potential role of GV in macrovascular complications.

## 6 GLUCOSE VARIABILITY AND PROMs

Clinicians and researchers often focus on hard clinical outcomes when assessing the role of glycaemic parameters. However, PROMs are equally important as changes in patient's QoL should also drive treatment decisions.

Reducing high glucose levels and avoiding hypoglycaemia appear to have a positive impact on QoL in individuals with diabetes^83^, ^84^ but the contribution of GV is less clear.

Earlier work on 60 T2D individuals suggested that the negative mood following meals is related to the rate of glucose excursion while another study of 23 T2D women showed that GV, measured as SD and CONGA, was associated with QoL measures.^85^, ^86^ A small study of 36 individuals with T1D, managed using insulin pumps,^87^ suggested that low mood is mainly related to high glucose levels rather than GV. However, it is difficult to generalise study findings given the limited patients studied, short period of CGM (48 h), overall good glycaemic control and use of insulin pumps in all participants. Another small study of 28 Japanese patients with T1D investigated the relationship between mean absolute glucose (MAG) and PROMs using 3 days of CGM data.^88^ PROMs included diabetes quality of life measures (DQOL) and diabetes treatment satisfaction questionnaire (DTSQ) with patients analysed in two groups of good/fair diabetes control (HbA1c <8%; *n* = 14) and inadequate control. A trend towards an inverse correlation was detected between MAG and DQOL (*r* = −0.35; *p* = 0.065), while MAG showed a significant negative correlation with DTSQ (*r* = −0.40; *p* = 0.034). These correlations were driven by the good/fair diabetes group, suggesting that high GV only affects PROMs in the presence of reasonable glycaemic control and larger studies are required to confirm these findings. A subsequent study of 57 people with T1D (20 on multiple daily insulin injections and 37 pump‐treated) failed to demonstrate a relationship between GV and DQOL.^89^ The authors were cautious in their interpretations and pointed out that the group studied may not be representative given the relatively good glycaemic control (HbA1c 7.9%) and the exclusion of those with a previous history of severe hypoglycaemia. Another possibility for the negative findings is related to the small number of patients studied and the use of DQOL only, which may have been inadequate on its own, and other questionnaires could have shown a difference.

A cross‐sectional study of 315 T1D patients has shown a very weak association between CoV and Pittsburgh Sleep Quality Index (PSQI) (*r* = 0.14; *p* = 0.03), although there was no difference between good and poor sleepers in relation to CoV or MAGE.^90^ However, GV measures were calculated using 7‐point capillary glucose testing, reducing confidence in study findings. A post hoc analysis of 139 T1D individuals from the GOLD trial demonstrated that reduction in HbA1c and increased time in range (TIR) were both associated with improved treatment satisfaction and reduced diabetes stress but GV (assessed as SD, CoV and MAGE) showed no such associations.^91^ The authors speculated that the failure to find associations may be related to patient focus on HbA1c, given the long diabetes duration is study participants, or that overall control is more important for healthy mental processes.

In another study of 60 T1D individuals,^92^ initiation of CGM improved both GV and PROMs but there was no relationship between the two. However, this study did not use common questionnaires, was conducted in a single centre and included mainly female patients, making generalisability of the findings difficult. A larger study of 312 individuals with T1D showed gender differences in QoL measures^93^ and also demonstrated that glycaemic instability, defined as the number of hypoglycaemic or hyperglycaemic episodes (<70 and >250 mg/dL) for 14 days prior to assessment, is an independent predictor of low QoL measures. However, this work used unconventional GV measures, and therefore, the relevance of findings is unclear. Another study of 249 T1D patients, including 83 individuals with a high score on patient health questionnaire‐9 (PHQ‐9), indicating a degree of depression, showed an association between low mood and inadequate diabetes control in general but there was no specific relationship with GV.^94^ In contrast, HbA1c variability has shown an association with PHQ‐9,^95^ and therefore, more targeted studies are required to fully understand the role of short‐term GV in depressive symptoms. Table 3 summarises the relationship between GV and PROMs.

**TABLE 3:** Summary of the association of patient‐reported outcomes with glycaemic variability (GV).

Study [ref]	Size (*n*)	Population	GV measure	Main findings
Cox et al., 2007^85^	33	T2D	Postprandial glucose	Low mood is associated with post‐prandial glucose excursions
Penckofer et al., 2012^86^	23	T2D	SD, CONGA	Greater GV may be associated with lower QoL and low moods
Hermanns et al., 2007^87^	36	T1D	Glucose AUC	Low mood is related to high glucose rather than glucose stability
Ayano‐Takahara et al., 2015^88^	28	T1D	MAG	MAG inversely correlated with DTSQ in people with better glucose control
Reddy et al., 2015^89^	57	T1D	CoV, SD, CONGA, LBGI, HBGI, MAGE, M‐value, MAG, MODD, ADRR	No correlation between GV and DQOL (the only PROM investigated)
Suteau et al., 2020,^90^	315	T1D	CoV, MAGE	CoV is weakly associated with PSQI and CoV (*r* = 0.14, *p* = 0.03)
Pylov et al., 2023^91^	139	T1D	SD, CoV, MAGE	None of the GV metrics is associated with treatment satisfaction
Castellano‐Guerrero et al., 2020^93^	312	T1D	Frequency of glucose <70 mg/dL or Frequency of glucose >250 mg/dL	Glycaemic instability (rather than GV) independently predicts low DQOL in females
Egbuonu et al., 2021^94^	249	T1D	SD and CoV	Inadequate diabetes control correlates with PHQ‐9 but GV metrics show no associations

Abbreviations: ADRR, average daily risk range; AUC, area under the curve; CONGA, continuous overlapping net glycaemic action; CoV, coefficient of variation; DQOL, diabetes quality of life; DTSQ, diabetes treatment satisfaction questionnaire; LBGI, low blood‐glucose index; MAG, mean absolute glucose; MAGE, mean amplitude of glycaemic excursion; MODD, mean of daily differences; PCI, percutaneous coronary intervention; PHQ‐9, patient health questionnaire‐9; SD, standard deviation of mean 24‐h glucose; T1D, type 1 diabetes; T2D, type 2 diabetes.

Taken together, studies linking GV and PROMs in diabetes are both limited and too small to draw definitive conclusions and future work in this area is required. Also, more attention should be given to analysing the independent effect of GV, away from hyperglycaemia and hypoglycaemia, to fully understand the role of glucose fluctuations in altering PROMs.

## 7 MECHANISMS OF GV‐MEDIATED RISK IN DIABETES

**FIGURE 2:**
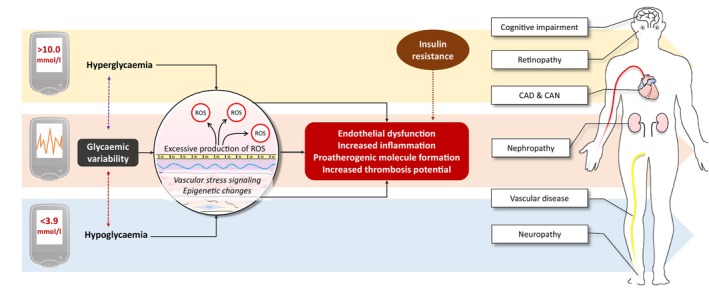
Potential mechanisms of glycaemic variability (GV)–induced vascular pathology. GV is associated with endothelial dysfunction, increased reactive oxygen species (ROS) production and epigenetic changes, creating an inflammatory and prothrombotic environment, thus contributing to vascular pathology. While current evidence strongly suggests an independent role for GV in vascular complications of diabetes, the interaction of GV metrics with both hypoglycaemia and hyperglycaemia, and even insulin resistance, can make disentangling the exact role of each problematic. Overall, it is likely that GV potentiates the adverse effects of metabolic abnormalities in diabetes, although the evidence for this remains largely circumstantial and more work in this area is required.

In common with hyperglycaemia and hypoglycaemia, potential mechanisms for GV‐mediated pathology have focused on the effects of oxidative stress and generation of reactive oxygen species (ROS) that are harmful to cells, particularly the endothelium (Figure 2). Markers of metabolic stress responses are elevated during postprandial periods and also during glucose swings, which correlate with MAGE.^96^ In vitro and in vivo studies attempted to dissect out the role of hyperglycaemia, hypoglycaemia and GV in these changes and suggested that GV has an independent additional effect.^97^, ^98^ This supports previous work showing that transient hyperglycaemia induces epigenetic changes in inflammatory molecules, thus promoting atherosclerosis.^99^ In an elegant study of 39 individuals with T2D, MAGE instability was associated with epigenetic changes in chromatin remodelling and impaired vascular function as measured by flow‐mediated dilation (FMD) of the brachial artery.^100^ This study does not only show the in vivo vascular effects for GV but also proposes interesting mechanisms.

Increased ROS correlates with higher MAGE and MODD, and, importantly, ROS decreased with improved GV in 68 individuals with T2D.^99^, ^101^ The association between oxidative stress and MAGE is evident even in younger, adolescent patients with diabetes (*n* = 34) and is particularly pronounced in those with T2D (*n* = 12),^102^ suggesting an interaction with insulin resistance. In addition to oxidative markers, the classical inflammatory marker C‐reactive protein (CRP) levels were raised with higher CGM‐derived SD in 17 adolescents with diabetes.^103^ Collectively, current evidence suggests that oscillation in glucose levels triggers atherogenic pathways more than persistent low or high glucose levels per se.

The effects of GV on vascular markers have been variable, which may be related to the population studied or the presence of other factors that enhance the vascular effects of GV. In support of this concept, GV was associated with a thrombotic environment in 107 individuals with T1D only in the presence of insulin resistance,^104^ suggesting an interaction between GV and insulin sensitivity.

## 8 MOVING FORWARD WITH GV: A PROPOSAL

Under normal physiological conditions, and despite daily activities that have the potential to induce large glucose swings, the human body keeps a tight glucose range and minimises fluctuations in glucose levels. This requires numerous interactions between different hormones and pathways, and therefore, maintaining low GV is likely to be important. This highly effective glucose control system is thrown into a disarray in diabetes, partly related to the pathophysiology of this condition and partly due to the therapies used.

**FIGURE 3:**
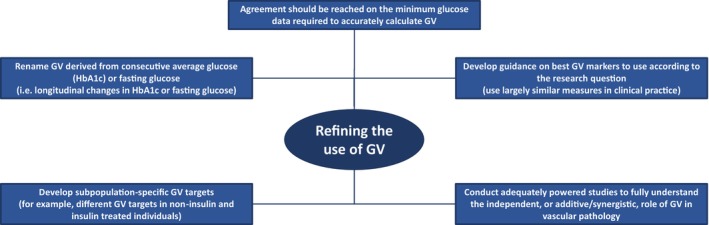
Refining the future use of glycaemic variability (GV). A number of steps should be taken to reduce the large number of GV markers in current use coupled with conducting appropriate studies to understand the independent, or additive/synergistic, role of GV in diabetes complications. This includes renaming GV markers that are not derived from frequent daily glucose measures, such as HbA1c and fasting glucose changes over a period of time. Findings from research studies should be translated into routine clinical use of different GV markers together with setting appropriate targets in the different subpopulations of people with diabetes.

There is little doubt that GV is one of the most difficult to understand glycaemic metrics. In addition to the large number of GV measures, the scientific community continues to present GV not only as variation in daily glucose levels but also as variation in average glycaemia, measured as HbA1c, as well variation in fasting glucose and even postprandial glucose. This further adds complexity to an area that is already confusing and therefore steps should be taken to simplify GV (Figure 3).

First, the scientific community needs to agree that adequate GV metrics can only be derived from frequent glucose checks, usually provided by CGM devices. Second, the aforementioned HbA1c, fasting and postprandial variability should be renamed and not called GV but corresponding changes of each of these glucose markers (for example, longitudinal changes in HbA1c). Third, an agreement should be reached on the main GV metrics to employ in future studies, as continuing the current trend of uncontrolled use is both confusing and counterproductive. In particular, studies should pre‐specify use of the GV metric(s) based on solid hypotheses rather than analysing multiple metrics and then deciding on those to report. Naturally, this does not mean that exploratory analysis cannot be performed (for hypothesis generation) but limiting the main analysis to a small number of metrics would reduce the risk of type 1 statistical errors. Fourth, we need good quality and adequately powered longitudinal studies to understand the exact contribution of GV to vascular complications of diabetes, and a possible direct effect on organ health (such as heart and brain) as well as potential effects on PROMs. Moreover, attempts should be made to dissect out the pathogenic role of GV from other glycaemic markers, particularly hyperglycaemia and hypoglycaemia, while also studying potential synergistic interactions between these glycaemic metrics, as well as other metabolic risk factors such as insulin resistance. Finally, from the clinical point of view, the current target for GV may lack ambition compared with other glycaemic markers. The most commonly used marker, CoV, is set at a target level over double that of individuals without diabetes, akin to setting an HbA1c target at 9%–10% (75–86 mmol/mol). While lowering CoV below 36% can be a challenge in well‐controlled MDI‐treated T1D patients,^105^ the increasing use of closed loop systems is clearly showing that lower targets can be reached. However, CoV can artificially increase with closed loop systems due to reduction in average glucose, highlighting the difficulties with GV assessment and emphasising the need to understand the appropriate use of different GV metrics. Also, consideration should be given to setting different targets to insulin and non‐insulin users as the latter group should easily achieve CoV <30%, even lower, and more work in this area is required. Also, targets should be set for other GV markers that can be important clinically, such as MAGE that is more effective than CoV at assessing glucose excursions.

Managing GV is a complex process and varies from one diabetes individual to another due to differences in lifestyle, type and duration of diabetes, and therapy‐related differences, including inter‐individual variability in response to a particular treatment. However, there are some simple concepts that can be followed to reduce GV. In those with high GV due mainly to hypoglycaemia, the type of agents used need to be reviewed, such as replacing a sulphonylurea with other agents in T2D, altering insulin doses, types or regimens in insulin‐treated diabetes, or replacing insulin injections with pumps or hybrid closed loop systems in T1D. Naturally, education around hypoglycaemia, including precipitating factors, such as alcohol and exercise, should form part of the consultation. In those with raised GV due to high post‐prandial glucose, lifestyle modifications, such as attention to diet and exercise, may help, or treatment changes can be considered such as the introduction of glucagon‐like peptide 1 receptor agonist therapies. Overall, clear guidance is needed on managing GV once the type of metrics used and cut off values are agreed in the different subpopulations of people with diabetes.

## 9 CONCLUSIONS

There is no doubt that consistently high glucose levels predispose to diabetes complications and can affect QoL but it has become apparent that both hypoglycaemia and GV also contribute to vascular pathology and patient well‐being. However, the adverse independent effects of GV have been difficult to establish until recently, given the limited glucose data provided by capillary glucose testing and difficulties in analysing potential interactions of GV with other glycaemic parameters. With the increased use of CGM, accumulating evidence indicates that GV can exert its deleterious vascular effects independently of other glycaemic markers, and therefore, more attention is needed to tackle GV in routine clinical practice. A key difficulty is the continued use of a large number of GV metrics, which is perhaps related to the complexity involved in evaluating this glycaemic parameter. It can be argued that CoV is currently regarded as the most clinically relevant GV marker but the target needs an update as having ‘one size fits all’ is perhaps too simplistic in a highly heterogeneous diabetes population. Moreover, while CoV is easy to understand and is objectively calculated, other GV metrics may be more sensitive in special circumstances.

Future longitudinal clinical studies are required to understand the relationship between GV and complications in different diabetes subpopulations with special focus placed on interactions with hypoglycaemia, hyperglycaemia and insulin resistance. In addition to hard clinical outcome studies, work is required to understand the relationship between GV and PROMs, an area that has been largely neglected and which may impact on QoL of people with diabetes.

## CONFLICT OF INTEREST STATEMENT

The author reports institutional research grants, honoraria, education support or consulting fees from the Abbott Diabetes Care, AstraZeneca, Bayer, Boehringer Ingelheim, Bristol‐Myers Squibb, Eli Lilly, GlaxoSmithKline, Menarini Pharmaceuticals, Merck Sharp & Dohme and Novo Nordisk.

### PEER REVIEW

The peer review history for this article is available at https://www.webofscience.com/api/gateway/wos/peer‐review/10.1111/dom.16098.

## Supporting information


Data S1.

## Data Availability

Not applicable given this is a narrative review.
