# Supplementary material for: The clinical importance of measuring glycaemic variability: Utilising new metrics to optimise glycaemic control
Source: Diabetes Obes Metab. 2024 Dec 5;26(Suppl 7):3–16. doi: 10.1111/dom.16098 (PMC11646482; doi:10.1111/dom.16098)
Supplement: Supplementary file 1 — Data S1. [file DOM-26-3-s001.docx]

**Supplementary Table 1. Database search strategy**

| **Glycaemic variability** |
| --- |
| (“glycemic variability” OR “glycaemic variability” OR “glucose variability” OR “SD” OR “Coefficient of Variation” OR “CV” OR “MAGE” OR “MAG” OR “MODD” OR “CONGA” OR “GVP”)[Title/Abstract] AND |
| **Macrovascular and microvascular outcomes** |
| (“cardiovascular” OR “vascular” OR “microvascular” OR “macrovascular” OR “coronary artery disease” OR “myocardial infarction” OR “acute coronary syndrome” OR “cerebrovascular disease” OR “MACE” OR “peripheral vascular disease” OR “neuropathy” OR “retinopathy” OR ”nephropathy” OR “microalbuminuria”)[Title/Abstract] |
| **Patient-related outcomes** |
| (“PROs”, OR “PROMs”, OR “QoL”, OR “DQOL” OR “HFS”, OR “DTSQ”, OR “PAID”, OR “DDS”, OR “EQ-5D”, OR “WHO-5”, OR “PHQ”) |
